# A multi-product, multi-period vaccine supply chain network planning with injection centers and storage conditions

**DOI:** 10.1371/journal.pone.0352121

**Published:** 2026-07-09

**Authors:** Reyhane Heydarpour, Hadi Mokhtari, Saeed Dehnavi

**Affiliations:** 1 Department of Master of Business Administration, Faculty of Financial Sciences, Management and Entrepreneurship, University of Kashan, Kashan, Iran; 2 Department of Industrial Engineering, Faculty of Engineering, University of Kashan, Kashan, Iran; Istinye University: Istinye Universitesi, TÜRKIYE

## Abstract

This study develops a mixed-integer linear programming model for a multi-product, multi-period vaccine supply chain network that incorporates injection centers and heterogeneous storage conditions, including cold and ultra-cold refrigeration. The model captures key operational decisions such as distribution center location, vaccine allocation, and storage configuration under capacity and demand constraints, while ensuring no shortage at injection centers. To efficiently solve large-scale instances, a tailored genetic algorithm (GA) is proposed. The main achievements of this research are as follows. First, the proposed model provides an integrated framework that simultaneously considers multi-vaccine characteristics and storage requirements within a three-tier supply chain. Second, computational experiments demonstrate that the GA achieves high-quality solutions with very small optimality gaps compared to exact solutions obtained by GAMS for small and medium-sized problems. Third, the results show that while exact methods become computationally inefficient or infeasible for large-scale instances, the GA remains robust and capable of producing near-optimal solutions within reasonable computational time. Finally, sensitivity analysis confirms the consistency and validity of the model, showing that total cost increases logically with demand and cost parameters. These findings highlight the effectiveness and scalability of the proposed approach and demonstrate its applicability as a decision-support tool for policymakers and healthcare planners in managing complex vaccine distribution systems, particularly during large-scale public health emergencies.

## Introduction

In recent years, healthcare managers have used various tools and methods to support continuous improvement strategies. Significant results are observed in waste reduction, process quality improvement, and reduced waiting times.

One of the tools that has recently entered the healthcare system is supply chain management (SCM) [1]. SCM ensures the availability of medicines, vaccines, and healthcare products at affordable prices and high quality. Health supply chain is an ecosystem of organizations, people, technology, activities, information, and resources that must come together to efficiently guarantee the product’s flow from pharmaceutical factories to reaching the final patient [2]. Recent studies have further extended the concept of supply chain networks by incorporating circular and closed-loop structures, emphasizing the integration of forward and reverse flows as well as sustainability considerations in complex systems [3]. Supply chains not only deliver medicines and healthcare items to people but also provide healthcare system planners with crucial information on needs, demands, and consumptions. Over the past 15 years, supply chain concepts have gained a strategic position in health management [4]. On the other hand, healthcare SCM is a diverse field requiring much more attention than in the past. In addition, the management of perishable products in supply chains has attracted significant attention due to their time-sensitive nature and storage requirements, which increase the complexity of planning and distribution decisions [5]. Despite having plans during emergencies, many organizations find themselves unprepared, lacking equipment and effective mechanisms to sustain healthcare operations. SCM in healthcare greatly aids the flexibility of a medical institution during crisis scenarios. Like any other system in the real world, health systems also require supply chain networks to operate effectively. Supply chains in healthcare systems encompass various goods such as blood, medicine, vaccines, and similar items [6]. These supply chain networks must ensure access to these goods at the right time, at a fair price, and with appropriate quality at points of demand. Among these items, vaccines are considered one of the most powerful tools for preventing and controlling infectious diseases [7]. As a result of vaccine use, diseases like smallpox have been eradicated and the rates of other diseases such as polio or measles have significantly decreased in many parts of the world. While vaccination is an appropriate medical intervention program aimed at prevention and improvement, successful implementation is impossible without proper planning and ensuring adequate logistics and supply chain. The vaccine distribution network encompasses the flow of vaccines from production and distribution points to demand points and typically includes suppliers, producers, distributors, and customers. Planning this system provides the ability to manage vaccines effectively within the healthcare system specially in the case of emergency such as COVID-19.

The COVID-19 pandemic led to the fastest development and deployment of new vaccines in human history [8]. Many countries have seen high vaccine uptake, with nearly 45% of people in the United Kingdom and the United States fully vaccinated by mid-June 2021. However, in Australia, Japan, and Malaysia, less than 5% were fully vaccinated. Achieving herd immunity typically requires around 70−90% vaccination coverage. Vaccine hesitancy due to rare serious side effects is a significant barrier, varying greatly among countries. Strategies to increase uptake include community engagement, effective communication with hesitant individuals using credible sources, and improving flexible access to vaccination. Some countries are considering additional strategies like incentives due to emerging variants. Despite safe and effective vaccines being available with recent advancements in production and widespread access approvals, motivation for vaccine uptake remains crucial for successful vaccination programs. Public health experts agree that the best solution to control a pandemic is preventive vaccination. However, the sudden emergence of COVID-19 has necessitated reactive vaccination. Due to limited vaccine availability, priority is given to individuals at higher risk. Prioritizing vaccination strategies aims to minimize the impact of widespread infections, deaths, years of life lost, and costs. Various public health sectors in the United States use a mixed vaccination strategy that transitions from prioritization to non-prioritization. Multi-criteria decision-making methods can effectively prioritize vaccination programs in the vaccine logistics network. The unprecedented effects of the coronavirus have led to global attention on the development and distribution of safe and effective vaccines. Over 160 million people are infected worldwide, with nearly 3.5 million deaths. Long-term protective measures and quarantines have severe financial impacts on society. The rush to reduce COVID-19 fatalities has accelerated the approval of various vaccine candidates. While global efforts have focused on developing vaccines and evaluating their effectiveness, challenges in vaccine supply chain management continue to significantly impact vaccination programs. High COVID-19 mortality rates prompted researchers to explore various treatment methods from antiviral drugs to plasma therapy since the disease’s onset. However, due to limited resources or low efficacy, the effectiveness of these methods remained debatable, highlighting the need for a preventive and effective approach to control the pandemic. The unprecedented need for producing and distributing sufficient safe and effective vaccines for mass immunization against COVID-19 has stressed global vaccine networks [9,10]. Considering the financial and operational benefits associated with vaccines, increased storage and transportation rates could be among the initial changes in the supply chain. The main objectives include minimizing costs and ensuring the fair distribution of vaccines. In addition, decisions regarding equipment types, facility locations, warehouses, and distribution centers must be optimized to efficiently meet demand across different supply chain nodes.

The rest of the paper is organized as follows: Section 2 reviews the related literature. Section 3 proposes the mathematical formulation of problem, and Section 4 develops the solving approach. Section 5 presents the analyses and results, and Section 6 concludes the paper.

## Literature review

In the past decade, the healthcare sector has undergone rapid changes. Due to increased competition, the growing influence of patient associations, and the necessity to deliver healthcare services more efficiently and effectively, many healthcare organizations have initiated projects focused on patient logistics, clinical pathways, data exchange, and vertical integration [11]. Furthermore, the redesign of hospital services and the implementation of integrated care programs are often regarded as key strategies for reducing resource utilization and improving the quality of healthcare. It is evident that the field of healthcare services has significantly transformed not only in practice but also in theory. Over the last ten years, a substantial number of studies across various domains such as economics, organizational behavior, and logistics have greatly enhanced our understanding of the healthcare sector [1]. Recent studies have emphasized the growing role of digitalization and technological support systems in improving transparency, traceability, and efficiency in vaccine supply chains [12].

The efficiency of vaccine supply chains is influenced by supply and demand, which has garnered the attention of operations management researchers. Key reasons for supply shortages include production uncertainties and insufficient incentives for manufacturers [13]. Successful immunization requires an effective vaccine supply chain that includes vaccine manufacturers to ensure vaccination for end users, particularly infants and pregnant mothers [6]. Duijzer et al. [14] reviewed the literature related to the vaccine supply chain. They categorized these studies by combining the World Health Organization’s priorities to create a strong and flexible vaccine supply chain with an operations research perspective, organizing them into groups focused on goods, production, allocation, and distribution modeling. De Boeck, et al. [4] examined vaccine distribution studies in low- and middle-income countries. They identified several issues that had received little or no attention in the operations research literature. Their findings indicated that operations researchers tend to focus heavily on strategic decisions within vaccine distribution chains while giving somewhat less emphasis to tactical and operational decisions. Hovav and Tsadikovich [15] utilized mathematical programming to propose an optimization model for designing a healthcare supply chain aimed at controlling the distribution of influenza vaccines and managing outbreaks effectively. Saif and Elhedhli [10] developed a cold supply chain for the distribution and inventory management of vaccines, taking environmental issues into account. To achieve this, they proposed a mixed-integer programming model aimed at minimizing total costs and employed a Lagrangian decomposition algorithm to solve the proposed model. Lim et al. [16], considering location issues and constraints, suggested a mixed-integer linear programming model for redesigning a cold supply chain for vaccine distribution. They created a hybrid heuristic algorithm to tackle large-scale problems and validated their model using data from several African countries. In addition, other studies have incorporated uncertainty and advanced predictive techniques such as machine learning into vaccine supply chain optimization models to enhance decision-making under demand variability [17]. Lin et al. [18] presented a model to address this issue by implementing inspection strategies. Their proposed chain includes distributors and retailers, analyzing transportation inspection policies for vaccines within the cold supply chain. Bulula et al. [9] utilized a cost breakdown method to analyze expenses in the vaccine supply chain, demonstrating that transferring responsibilities from medical stores to premium providers resulted in a 27% reduction in distribution and storage costs for vaccines. Enayati and Özaltın [19] introduced a mathematical programming model aimed at equitable distribution of influenza vaccines, dividing the population into several subgroups and fairly allocating necessary vaccines to each subgroup to prevent epidemic outbreaks. Rastegar et al. [20] took a step further by addressing the issue of location inventory during the COVID-19 pandemic. They developed a mixed-integer linear programming model for the equitable distribution of influenza vaccines. This research considers practical assumptions such as storage capacity for future periods, shortages, and budget constraints. Recent research has also focused on resilience in vaccine supply chains, particularly highlighting structural weaknesses exposed during the COVID-19 pandemic and proposing strategies for improving robustness and adaptability [21]. Moreover, recent optimization studies have addressed multi-period vaccine supply chain network design under uncertainty, incorporating capacity expansion and stochastic demand considerations [22]. Recent studies have further developed multi-period and multi-echelon vaccine supply chain network design models under uncertainty, incorporating capacity expansion and stochastic demand considerations [23].

With the widespread rollout of vaccines, employers are seeking ways to encourage their employees to get vaccinated. The U.S. Equal Employment Opportunity Commission has issued guidelines confirming that employers can offer incentives to employees who voluntarily receive the COVID-19 vaccine. Additionally, employees who voluntarily confirm that they or their family members have been vaccinated will also be eligible for incentives [24]. For example, Aldine Independent School District is offering a $500 incentive to eligible full-time employees, permanent part-time staff, dedicated substitutes, and long-term substitutes serving as teachers, all while prioritizing the safety and well-being of staff and students. Furthermore, eligible individuals must be fully vaccinated against COVID-19. It is worth noting that if an incentive scheme incurs significant costs or damages and does not effectively increase vaccine uptake, it cannot be justified. On the other hand, financial incentives are among the interventions that are likely to have a positive impact on vaccine uptake. Evidence from successful incentive programs for hepatitis B and influenza suggests that financial incentives could be beneficial in promoting adherence to COVID-19 vaccinations. Similarly, Germany has successfully implemented a payment system for plasma donation to increase supply and address shortages. However, a survey study with 1,349 participants found that payments of up to 200 euros did not increase the willingness to receive the COVID-19 vaccine [8]. More recently, many researchers considered different aspect of management under COVID-19 [25].

Recent developments in optimization, stochastic systems, control theory, and computational intelligence have significantly expanded the applicability of mathematical methods in healthcare logistics and supply chain planning. In particular, Kropat et al. [26] investigated optimization and homogenization techniques for complex dynamic network systems and large-scale operational structures, highlighting the role of advanced mathematical optimization in computationally difficult systems. Similarly, Savku & Weber [27] and Savku [28] proposed several stochastic control and regime-switching models involving delay, memory, and stochastic differential games, demonstrating the applicability of stochastic optimization and dynamic control approaches in uncertain decision environments.

Yerlikaya-Özkurt et al. [29] contributed to optimization and numerical modeling approaches in applied mathematical systems, especially under uncertainty and dynamic conditions. Özmen & Weber [30] also investigated robust optimization and intelligent computational methods for complex operational and engineering systems, emphasizing the importance of uncertainty-aware decision frameworks in optimization problems. In addition, Pakize Taylan studied statistical and computational modeling approaches for uncertain systems and forecasting structures, which are highly relevant to healthcare demand estimation and logistics planning.

Kuter et al. [31] focused on dynamic optimization and operational analytics for engineering and management systems. Their studies highlighted the importance of integrating computational intelligence with operational research methodologies in large-scale planning environments. Similarly, Özöğür-Akyüz and Weber [32] investigated decision-support methodologies and optimization-based approaches for complex industrial and logistics systems, emphasizing resilience and adaptive planning strategies under uncertain operational conditions.

Furthermore, Kropat et al. [33] contributed to uncertainty-based regression and optimization models for dynamic systems, particularly through set-theoretic and ellipsoidal optimization approaches in network environments. Gürbüz et al. [34] investigated optimization and analytical approaches in industrial engineering and operational systems, demonstrating the effectiveness of mathematical programming in resource allocation and system efficiency improvement.

Research conducted by Yılmaz et al. [35] also emphasized dynamic system behavior, intelligent optimization, and computational methods in engineering applications. In addition, Hamidoğlu and Weber [36] explored optimization-based analytical frameworks and stochastic modeling techniques for agricultural systems.

Moreover, Baltas, et al. [37] contributed to recent developments in sustainable logistics, supply chain optimization, computational intelligence, and operational planning. Their studies highlighted the growing importance of integrating metaheuristic algorithms, stochastic optimization, and resilient supply chain structures into modern operational research frameworks for complex real-world systems.

Motivated by these developments, the present study integrates mathematical optimization and metaheuristic solution methodologies into a multi-product and multi-period vaccine supply chain framework. Unlike many existing studies that focus only on epidemic dynamics or inventory policies, the proposed model simultaneously considers facility location, heterogeneous refrigeration requirements, and vaccine allocation decisions within an integrated optimization framework.

The proposed model is a large-scale, multi-period, multi-product mixed-integer programming formulation, which inherently leads to high computational complexity due to the simultaneous consideration of strategic (facility location and storage configuration), tactical (allocation decisions), and operational (flow and distribution) decisions. The inclusion of multiple vaccine types with different storage requirements (cold and ultra-cold), along with capacity constraints, binary facility-opening decisions, and time-dependent demand across injection centers, significantly increases the dimensionality and combinatorial nature of the problem. As a result, the model becomes NP-hard, and its exact solution time grows rapidly with problem size, as also observed in the computational experiments where GAMS fails to solve the largest instances ().

In terms of superiority, the proposed model offers a more comprehensive and realistic representation of vaccine supply chain networks compared to existing studies. Unlike most previous works that focus on single-product or single-period settings, the model integrates multi-product flows, heterogeneous storage conditions, and a three-tier structure (producers, distribution centers, and injection centers) within a unified framework. Additionally, the model explicitly incorporates storage technology decisions (cold vs. ultra-cold), which is a critical requirement in real-world vaccine logistics, especially during pandemic conditions.

Furthermore, the development of a tailored genetic algorithm significantly enhances the solvability of the model for large-scale instances. The computational results demonstrate that the GA consistently produces near-optimal solutions with very small optimality gaps compared to exact solutions for small and medium cases, while remaining computationally efficient when exact methods become infeasible. Sensitivity analysis also confirms the stability and logical behavior of the model under varying demand and cost conditions, reinforcing its practical robustness.

## Methodology

### Problem description

Vaccine supply chain is considered to be three-tiered and multi-period, including production centers, distribution centers equipped with cold and ultra-cold refrigerators, and injection centers. The current supply chain considers multiple products and includes three types of vaccines, each requiring specific storage conditions involving cold and ultra-cold refrigeration. Cold and ultra-cold refrigerators can be installed at distribution centers.

Assumptions of the problem are:

- The proposed model is multi-product and multi-period;
- A three-tier supply chain is considered with vaccine producers, distribution centers, and injection centers;
- The model determines the location of distribution centers;
- Established distribution centers can manage refrigerated and ultra-cold storage for vaccines;
- Each producer can place one order in each period;
- Distribution centers have capacities;
- Each producer produces only one type of vaccine;
- Each distribution center can be equipped simultaneously with refrigerated and ultra-cold storage or one of them;
- Vaccine shortage at injection centers is not allowed.

### Notations

**Indices:**

w Index of production centers, w=1,2,…,W.

j Index of vaccines, j=1,2,…,J.

c Index of distribution centers, c=1,2,…,V.

s Index of injection centers, s=1,2,…,S.

t Index of time periods, t=1,2,…,T.

**Parameters:**

${TD}_{jst}$ The demand for vaccine j in injection center s at period t

${VX}_{j}$ 1 if vaccine j needs cold refrigerator, and 0 if needs ultra-cold refrigerator (a binary parameter)

$\overset{M}{\underset{wjt}{EX}}$ The cost of ordering vaccine j from producer w at period t

$\overset{SD}{\underset{ct}{EX}}$ The fixed opening cost of distribution center *c* equipped with a cold refrigerator at period t

$\overset{SD}{\underset{ct}{EY}}$ The fixed opening cost of distribution center c equipped with an ultra-cold refrigerator at period t

${PV}_{wjt}$ The purchasing cost of vaccine j from producer w at period t

$\overset{M}{\underset{wjct}{TV}}$ The transportation cost of vaccine j from producer w to distribution center c at period t

$\overset{SD}{\underset{jcst}{TV}}$ The transportation cost of vaccine j from distribution center c to injection center s at period t

${HXV}_{jct}$ The holding/storing cost of vaccine j at distribution center c which is equipped with a cold refrigerator at period t

${HYV}_{jct}$ The holding/storing cost of vaccine *j* at distribution center c which is equipped with an ultra-cold refrigerator at period t

$\overset{CR\;}{\underset{c}{MD}}$ The capacity of distribution center c for holding/storing cold vaccines

$\overset{VCR}{\underset{c}{MD}}$ The capacity of distribution center c for holding/storing ultra-cold vaccines

M A big positive number

**Decision variables:**

${vdx}_{wjct}$ The number of cold vaccines j that are allocated from producer w to distribution center c at period t

${vdy}_{wjct}$ The number of ultra-cold vaccines j that are allocated from producer w to distribution center c at period t

$v_{jcst}$ The number of vaccines j that are transported from distribution center c to injection center s at period t

$\overset{SD}{\underset{c}{z}}$ 1 if the distribution center c is opened and 0, otherwise (A binary variable)

$\overset{SD}{\underset{c}{x}}$ 1 if the distribution center c is equipped with cold refrigerator and 0, otherwise (A binary variable)

$\overset{SD}{\underset{c}{y}}$ 1 if the distribution center c is equipped with ultra-cold refrigerator and 0, otherwise (A binary variable)

### Objective Function (Total Cost)

The cost of setting up a distribution center c equipped with a cold refrigerator in period t is represented by parameter $\overset{SD}{\underset{ct}{EX}}$. Additionally, if distribution center c equips with a cold refrigerator, it takes the value of one; otherwise, it takes the value of zero, denoted by variable $\overset{SD}{\underset{wc}{x}}$. Therefore, the product of the variable and cost parameter represents the cost of setting up distribution centers equipped with cold refrigerators. So, the total cost of setting up distribution centers equipped with cold refrigerators can be formulated as follows:


$$\begin{array}{c} \sum_{c}\sum_{t}\overset{SD}{\underset{ct}{EX}}\overset{SD}{\underset{c}{x}}. \end{array}$$ (1)

Similarly, the total cost of setting up distribution centers equipped with ultra-cold refrigerators is formulated as:


$$\begin{array}{c} \sum_{c}\sum_{t}\overset{SD}{\underset{ct}{EY}}\overset{SD}{\underset{c}{y}}. \end{array}$$ (2)

The cost of purchasing vaccine j in period t from producer w is shown by parameter ${PV}_{wjt}$. Additionally, the number of vaccines j (requiring regular cold refrigeration) allocated from producer w to distribution center c in period t is represented by parameter ${vdx}_{wjct}$. Furthermore, the number of vaccines j (requiring ultra-cold storage) allocated from producer w to distribution center c in period t is denoted by parameter ${vdy}_{wjct}$. Consequently, the product of two variables with the cost parameter represents the cost of purchasing vaccines from different producers. So, the total cost of purchasing vaccines from different producers can be calculated by:


$$\begin{array}{c} \sum_{w}\sum_{j}\sum_{c}\sum_{t}{PV}_{wjt}{(vdx}_{wjct}+{vdy}_{wjct}). \end{array}$$ (3)

The cost of transporting vaccines j from producer w to distribution center c in period t is represented by the parameter $\overset{M}{\underset{wjct}{TV}}$. Additionally, the number of vaccines j (requiring regular refrigeration) allocated from producer w to distribution center c in period t is denoted by the parameter ${vdx}_{wjct}$. Furthermore, the number of vaccines j (requiring ultra-cold refrigeration) allocated from producer w to distribution center c in period t is indicated by the parameter ${vdy}_{wjct}$. Therefore, the product of two variables multiplied by the cost parameter represents the cost of transporting vaccines from the producer to distribution centers. Hence, the total cost of transporting vaccines from the producer to distribution centers can be stated as:


$$\begin{array}{c} \sum_{w}\sum_{j}\sum_{c}\sum_{t}\overset{M}{\underset{wjct}{TV}}{(vdx}_{wjct}+{vdy}_{wjct}). \end{array}$$ (4)

The cost of transporting vaccines j from distribution center c to injection center s in period t is represented by the parameter $\overset{SD}{\underset{jcst}{TV}}$. Additionally, the number of vaccines j sent from distribution centers c to injection centers s in period t is denoted by the variable $v_{jtcs}$. Therefore, the product of this variable and the cost parameter represents the cost of transporting vaccines from distribution centers to injection centers. So, the total cost of transporting vaccines from distribution centers to injection centers can be computed by:


$$\begin{array}{c} \sum_{j}\sum_{c}\sum_{s}\sum_{t}\overset{SD}{\underset{jcst}{TV}}v_{jtcs}. \end{array}$$ (5)

The cost of storing vaccine j at distribution center c equipped with a cold refrigerator in period t is represented by the parameter ${HXV}_{jct}$. Additionally, the number of vaccines j (requiring a cold refrigerator) allocated from producer w to distribution center c in period t is denoted by the variable ${vdx}_{jct}$. Therefore, the product of the variable and cost parameter represents the cost of storing vaccines at distribution centers equipped with cold refrigerators. Therefore, the total cost of storing vaccines at distribution centers equipped with cold refrigerators is as follows:


$$\begin{array}{c} \sum_{j}\sum_{c}\sum_{t}{HXV}_{jct}{vdx}_{jct}. \end{array}$$ (6)

In a similar way, the total cost of storing vaccines at distribution centers equipped with ultra-cold refrigerators is:


$$\begin{array}{c} \sum_{j}\sum_{c}\sum_{t}{HYV}_{jct}{vdy}_{jct}. \end{array}$$ (7)

The total objective function, which is the sum of all of the above costs, is presented below:


$$\begin{array}{cc} \text{min}TC= & \sum_{c}\sum_{t}\overset{SD}{\underset{ct}{EX}}\overset{SD}{\underset{c}{x}}+\sum_{c}\sum_{t}\overset{SD}{\underset{ct}{EY}}\overset{SD}{\underset{c}{y}}+\sum_{w}\sum_{j}\sum_{c}\sum_{t}{PV}_{wjt}{(vdx}_{wjct}+{vdy}_{wjct})\\ & +\sum_{w}\sum_{j}\sum_{c}\sum_{t}\overset{M}{\underset{wjct}{TV}}{(vdx}_{wjct}+{vdy}_{wjct})+\sum_{j}\sum_{c}\sum_{s}\sum_{t}\overset{SD}{\underset{jcst}{TV}}v_{jtcs}\\ & +\sum_{j}\sum_{c}\sum_{t}{HXV}_{jct}{vdx}_{jct}+\sum_{j}\sum_{c}\sum_{t}{HYV}_{jct}{vdy}_{jct}. \end{array}$$ (8)

### Constraints

The constraint (9) ensures that if vaccine j requires cold storage equipment until refrigeration equipment is established, it will not receive an order from production centers:


$$\begin{array}{c} {vdx}_{wjct}\leq M\times {VX}_{j},\forall w,j,c,t. \end{array}$$ (9)

According to constraint (10), if vaccine j requires very cold storage equipment until ultra-cold refrigeration is established, it will not receive an order from production centers:


$$\begin{array}{c} {vdy}_{wjct}\leq M\times \left({\text{1}-VX}_{j}\right),\forall w,j,c,t. \end{array}$$ (10)

The constraint (11) states that more vaccines are not allocated beyond the capacity of distribution centers equipped with cold refrigerators, as follows:


$$\begin{array}{c} \sum_{w,j}{vdx}_{wjct}\leq \overset{CR}{\underset{c}{MD}},\forall c,t. \end{array}$$ (11)

The constraint (12) states that more vaccines are not allocated beyond the capacity of distribution centers equipped with ultra-cold refrigerators:


$$\begin{array}{c} \sum_{w,j}{vdy}_{wjct}\leq \overset{VCR}{\underset{c}{MD}},\forall c,t. \end{array}$$ (12)

The constraint (13) indicates that if a distribution center without suitable equipment for vaccines requiring refrigeration is not set up, it will not receive an order from production centers:


$$\begin{array}{c} \sum_{j,t}{vdx}_{wjct}\leq M.\overset{SD}{\underset{wc}{x}},\forall w,c. \end{array}$$ (13)

The constraint (14) indicates that if a distribution center without suitable equipment for vaccines requiring ultra-cold refrigeration is not set up, it will not receive an order from production centers:


$$\begin{array}{c} \sum_{j,t}{vdy}_{wjct}\leq M.\overset{SD}{\underset{wc}{y}},\forall w,c. \end{array}$$ (14)

The constraint (15) guarantees that if a distribution center is opened, then it can be equipped with cold and ultra-cold refrigerators:


$$\begin{array}{c} \overset{SD}{\underset{c}{x}}+\overset{SD}{\underset{c}{y}}\leq \text{2}\overset{SD}{\underset{c}{z}},\forall c. \end{array}$$ (15)

The number of vaccines delivered to each distribution center in each period is calculated by Equation (16), meaning each distribution center, if established, can only receive service from one production center:


$$\begin{array}{c} \sum_{w}\left({vdx}_{wjct}+{vdy}_{wjct}\right)=\sum_{s}v_{jcst},\forall j,c,t. \end{array}$$ (16)

Equation (17) guarantees that demand at injection centers equals the total vaccines transferred from distribution centers to injection centers:


$$\begin{array}{c} {TD}_{jst}=\sum_{c}v_{jcst},\forall j,s,t. \end{array}$$ (17)

## GA-based Solution

In order to illustrate the performance of the proposed mathematical programming approach for the vaccine supply chain planning problem, three different analyses are conducted in this section. First, an illustrative example is presented and analyzed in detail to demonstrate the structure and functionality of the proposed model. Second, a set of computational experiments with small-, medium-, and large-sized problem instances is performed to evaluate the efficiency and solution quality of the proposed genetic algorithm compared to exact solutions obtained by GAMS. Third, sensitivity analyses are carried out to examine the impact of key parameters on the objective function and to validate the robustness of the model under different conditions.

### Chromosome representation

**Fig 1:**
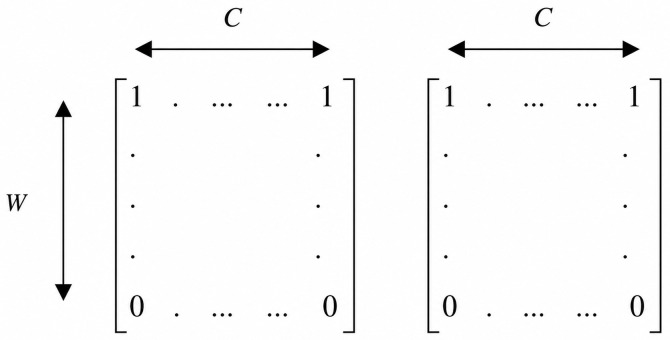
The chromosome structure.

In the current algorithm, the chromosome essentially represents the solution related to the binary variables of the problem. This allows for a quick understanding of the allocation status of production centers to distribution centers, as well as whether the refrigerator at the specified distribution center is set to cold or ultra-cold. The general form of the chromosome in question is illustrated in the Fig 1.

As shown in the figure above, the structure of the chromosome consists of two matrices positioned adjacent to each other, where the row index represents production centers and the column index represents distribution centers. If any entry has a value of one, it indicates that the corresponding production center (represented by the row) is allocated to the corresponding distribution center (represented by the column). Additionally, the first matrix illustrates allocations related to cold storage, while the second matrix depicts allocations for very cold storage.

### Initial solution

**Fig 2:**
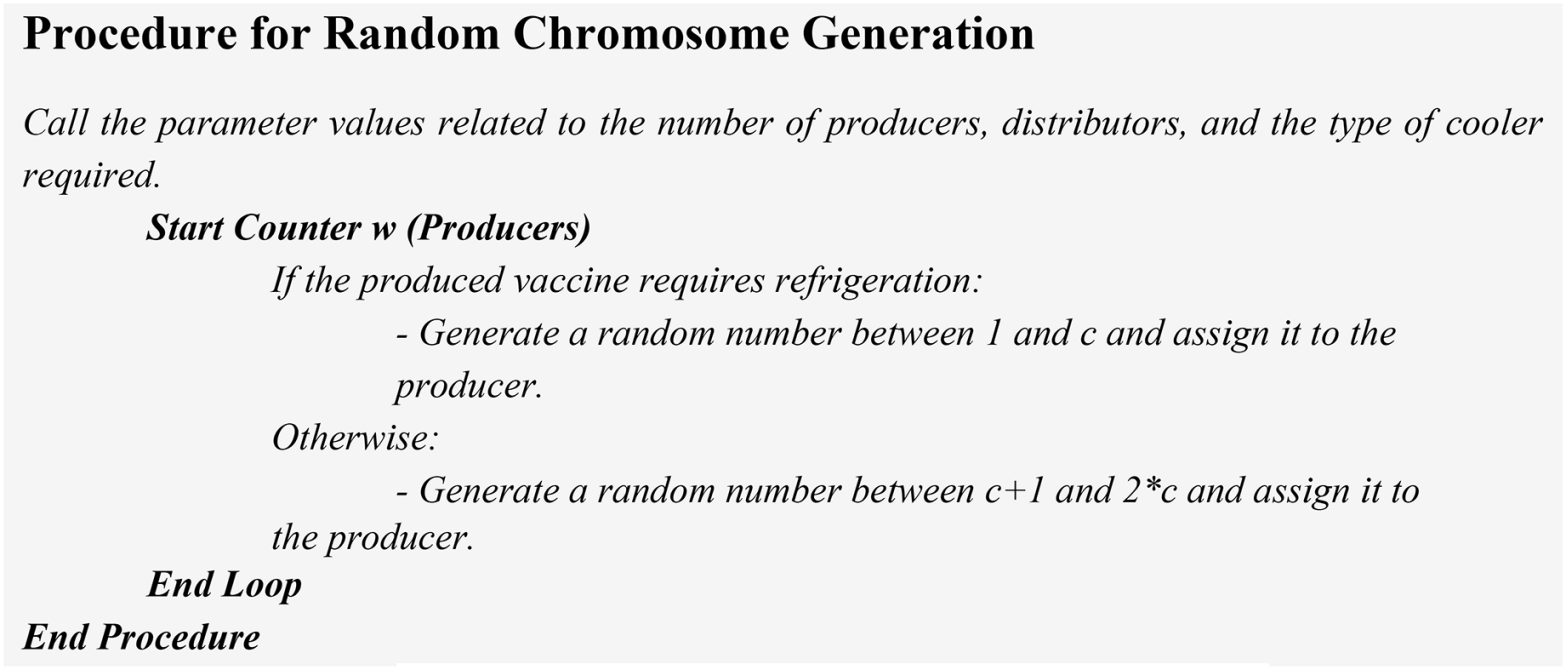
Initial solution generating scheme.

The initial solutions must be generated in such a way that, while being reasonable, they are as close as possible to the final optimal solution. To achieve this, the procedure shown in Fig 2 has been utilized in the genetic algorithm programming:

The above function is designed to generate initial chromosomes. To achieve this, an empty chromosome matrix is created at the beginning of the program. Then, within the corresponding loop, a count of producers is conducted, and each producer is randomly assigned to distribution centers with the required refrigeration conditions, either cold or very cold, based on their vaccine production needs. To prevent the generation of invalid solutions, at the start of the loop, a check is performed to ensure that the required refrigeration system for each produced vaccine is either cold or ultra-cold, thereby avoiding incorrect allocations to inappropriate cooling systems.

### Reverse planning

**Fig 3:**
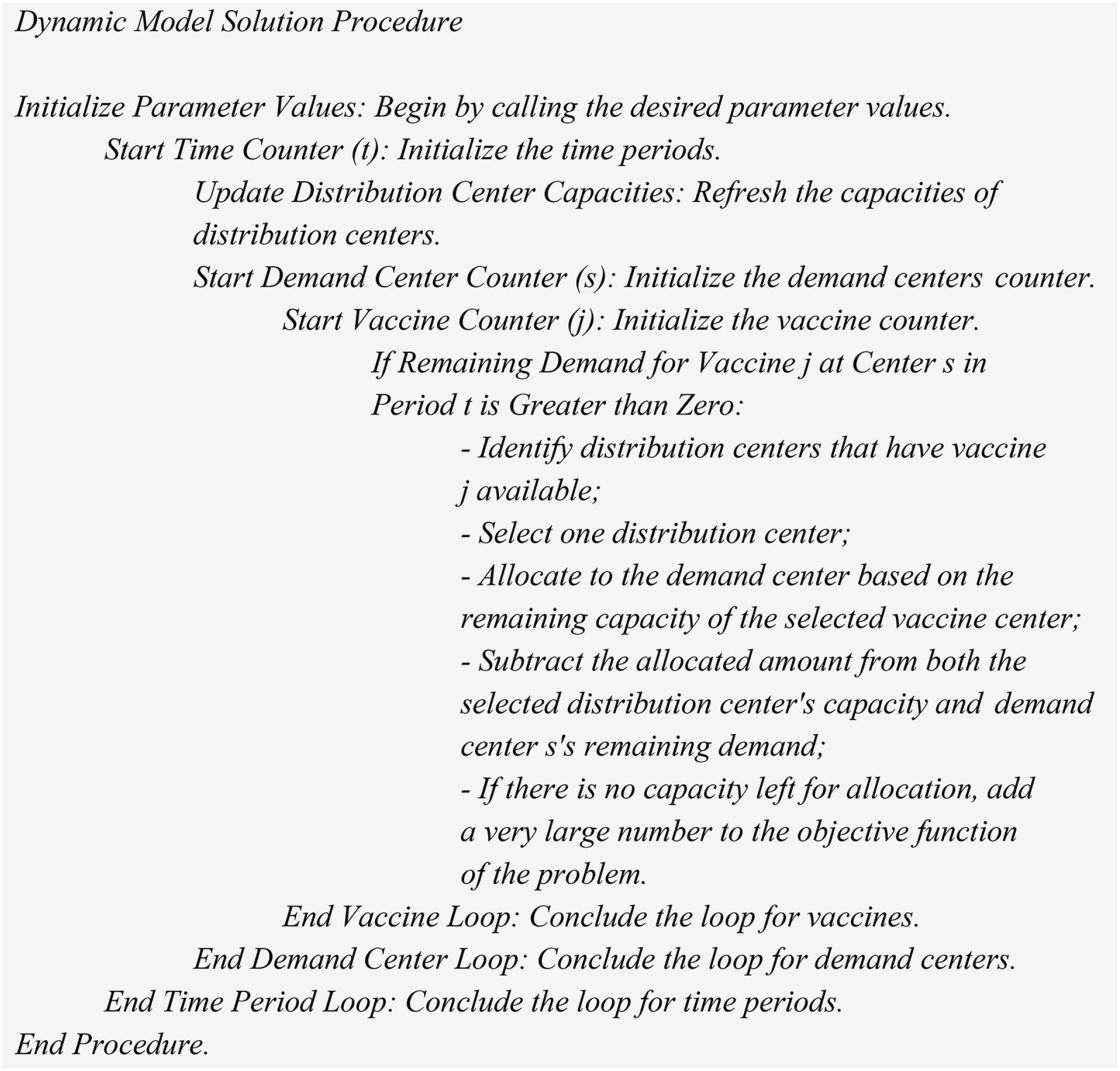
Reverse solution approach.

After generating the chromosome and determining the allocation method for distributing centers to vaccine production facilities, the next step is to solve the allocation-based model and obtain the flow of items across different layers of the supply chain. To achieve this, a reverse dynamic programming approach will be utilized, starting from the end of the supply chain, specifically at the demand centers. The procedure and programming code for solving the model are outlined as presented by Fig 3.

### Crossover

**Fig 4:**
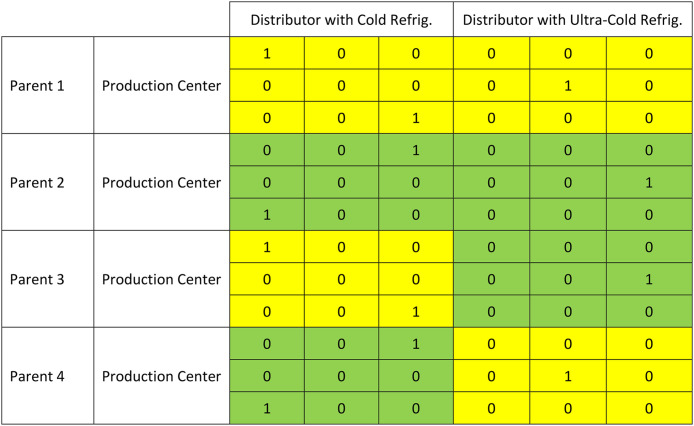
Crossover illustration.

In the previous sections, the method for generating random solutions in the first generation and solving the model based on the chromosome produced in each generation was explained. To move towards an optimal solution in genetic algorithms, the chromosomes of each generation must undergo operations that produce new chromosomes for the next generation. This section will describe the implementation of the crossover operator to generate new solutions. Fig 4 shows the crossover procedure.

As illustrated in the figure, to generate a new solution using the crossover operator, two chromosomes are randomly selected as parents. The first offspring is created by combining the first half of the first parent chromosome with the second half of the second parent chromosome. Similarly, the second offspring is generated using the reverse halves of the parents. Next, we utilize the previously introduced solve function to determine the values of the new chromosomes, and ultimately, we calculate each offspring’s objective function.

### Mutation

**Fig 5:**
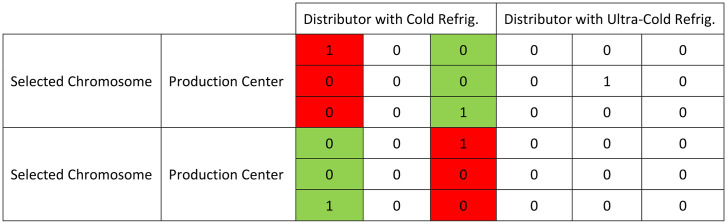
Mutation illustration.

To implement the mutation operator, a chromosome is randomly selected, and two columns of its corresponding matrix are swapped. The new chromosome is then directed to the solve function, where the values of the decision variables are calculated, and its objective function value is determined. The Fig 5 schematically illustrates how this operator is implemented.

### Pseudo-code

To enhance the clarity and reproducibility of the proposed solution approach, a structured pseudo-code of the developed Genetic Algorithm (GA) is presented below. This pseudo-code outlines the main steps of the algorithm, including initial population generation, fitness evaluation based on the reverse allocation procedure, and the application of genetic operators such as selection, crossover, and mutation. It also highlights how feasibility is maintained with respect to storage conditions and capacity constraints. The proposed GA is specifically tailored to efficiently handle the complexity of the multi-product, multi-period vaccine supply chain network design problem.

Genetic Algorithm for Vaccine Supply Chain Optimization

1. Initialize parameters:

- Population size (PopSize);

- Maximum number of generations (MaxGen);

- Crossover rate (Pc);

- Mutation rate (Pm).

2. Generate initial population:

For each chromosome in population:

- Randomly assign producers to distribution centers;

- Ensure feasibility based on storage type (cold/ ultra-cold).

3. Evaluate fitness:

For each chromosome:

- Decode chromosome;

- Apply reverse allocation procedure;

- Compute total cost (objective function).

4. Repeat until termination condition (MaxGen reached):

4.1 Selection:

- Select parent chromosomes based on fitness (e.g., roulette wheel or tournament).

4.2 Crossover:

- With probability Pc;

- Select two parents;

- Exchange partial matrices to create offspring.

4.3 Mutation:

- With probability Pm;

- Randomly select a chromosome;

- Swap two columns in allocation matrix.

4.4 Repair (if needed):

- Ensure feasibility of offspring (storage compatibility, capacity constraints).

4.5 Evaluation:

- Compute fitness of new chromosomes using reverse solution procedure.

4.6 Replacement:

- Form new population (elitism can be applied).

5. Output:

- Best chromosome found;

- Corresponding objective function value.

## Computational results

In this section, we will explore the solution of models in various dimensions using GAMS and genetic algorithms, followed by a comparison of the results. We will present different examples for solving the model across three categories: small, medium, and large dimensions. The problems will be solved using both precise methods with GAMS (where feasible) and a genetic algorithm implemented in MATLAB. The comparison of the obtained results will demonstrate the capability of the developed genetic algorithm for the problem at hand.

### Small Sized Problems

An example with small dimensions was solved using a small-scale model, which involved six problems. The dimensions of each problem, along with the time taken and the results obtained in both software programs, are detailed by the Table 1.

**Table 1:** The solution results for small problems.

Problem Dimension	Example Number	Number of								Solution Approach						
		Production Center	Vaccine	Distribution Center	Injection Center	Time Period	Binary Variable	Integer Variable	Constraint	GAMS		GA				Gap%
										Optimal Objective Function	CPU Time (s)	Population Size	Number of Generation	Best Objective Function	Standard Deviation	
**Small-sized**	1	3	3	3	3	3	21	243	147	51389	2	50	20	51389	254.21	0
	2	5	5	5	5	3	55	1125	385	148890	3	50	50	148890	585.3	0
	3	10	10	8	6	5	168	10400	1748	536433	5	200	100	705823	956.8	0.3158
	4	10	10	10	6	5	210	13000	2110	116384	10	500	150	116476	568.5	0.0008
	5	12	12	12	8	5	300	23040	3060	157215	13	500	150	158690	652.1	0.0094
	6	14	14	14	6	2	406	13328	1806	107478	17	500	150	118020	595.2	0.0981

**Fig 6:**
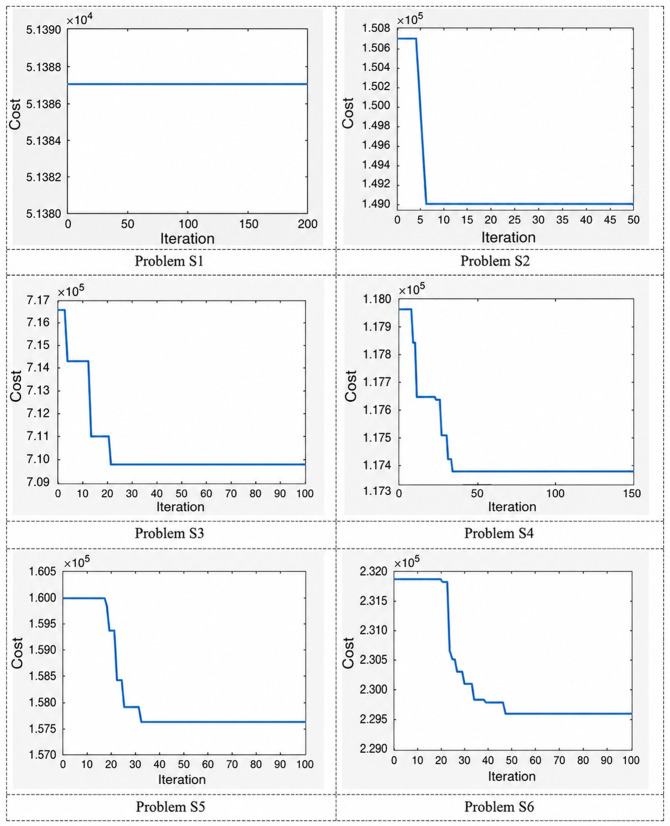
The convergence curve for small problems.

In Fig 6, the convergence chart related to the metaheuristic best solution for each of the above cases is presented.

### Medium sized problems

In this section, the mathematical model of the problem is solved with dimensions slightly larger than those of the smaller dimensions. However, it is still possible to solve all these dimensions using the GAMS software. Therefore, as in the previous case, the model is solved using both GAMS and MATLAB, and the results will be compared in terms of solution values and computation time. The results obtained from solving six problems of medium dimensions are presented in the Table 2.

**Table 2:** The solution results for medium problems.

Problem Dimension	Example Number	Number of								Solution Approach						
		Production Center	Vaccine	Distribution Center	Injection Center	Time Period	Binary Variable	Integer Variable	Constraint	GAMS		GA				Gap%
										Optimal Objective Function	CPU Time (s)	Population Size	Number of Generation	Best Objective Function	Standard Deviation	
**Medium-sized**	7	14	14	14	6	2	406	13328	1806	53739	20	500	150	54345	421.2	0.0113
	8	14	14	14	12	2	406	15680	1974	96953	24	500	150	98767	658.0	0.0187
	9	14	14	14	8	3	406	21168	2590	101483	35	500	150	103436	689.6	0.0192
	10	14	14	14	12	3	406	23520	2758	137815	52	1000	200	144641	798.65	0.0495
	11	16	16	16	12	3	528	33792	3504	168348	189	1000	200	168465	954.6	0.0007
	12	16	16	16	14	3	528	35328	3600	194780	421	1000	250	196209	990.5	0.0073

**Fig 7:**
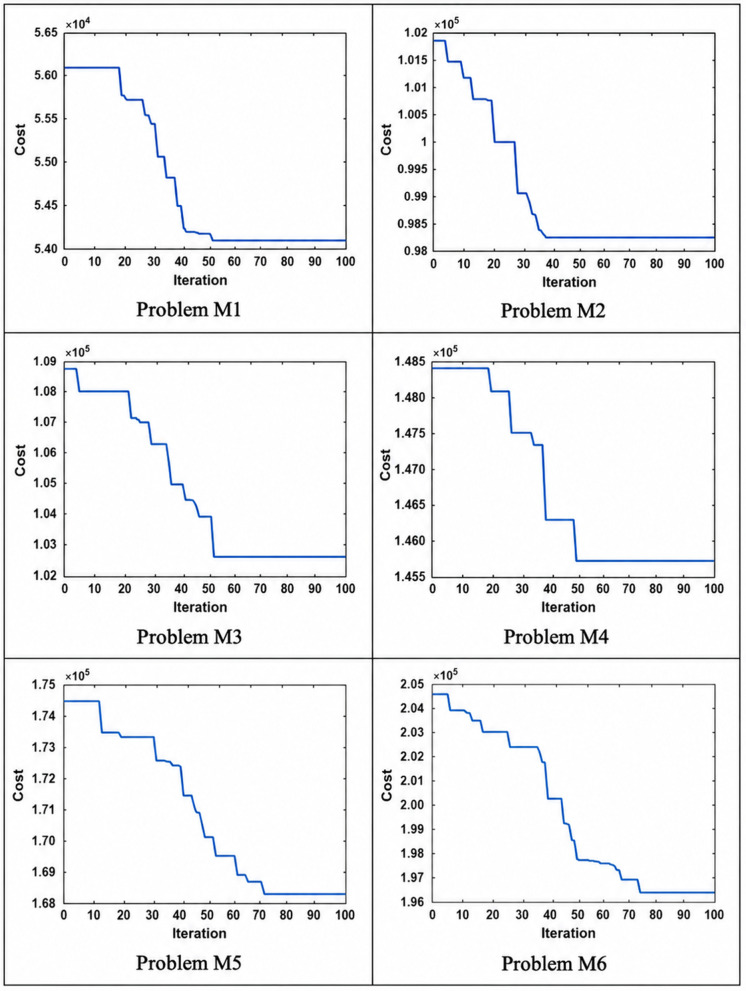
The convergence curve for medium problems.

As the results from solving the model in medium dimensions indicate, the genetic algorithm continues to provide solutions with a small gap and close to optimal, even in medium dimensions where GAMS gradually requires significantly more time to solve and the complexity of the problem increases considerably. This suggests that the genetic algorithm has been designed and developed to be highly efficient for this problem. Fig 7 shows the convergence graph for each of the six scenarios of solving the model in medium dimensions using the genetic algorithm.

### Large sized problems

**Fig 8:**
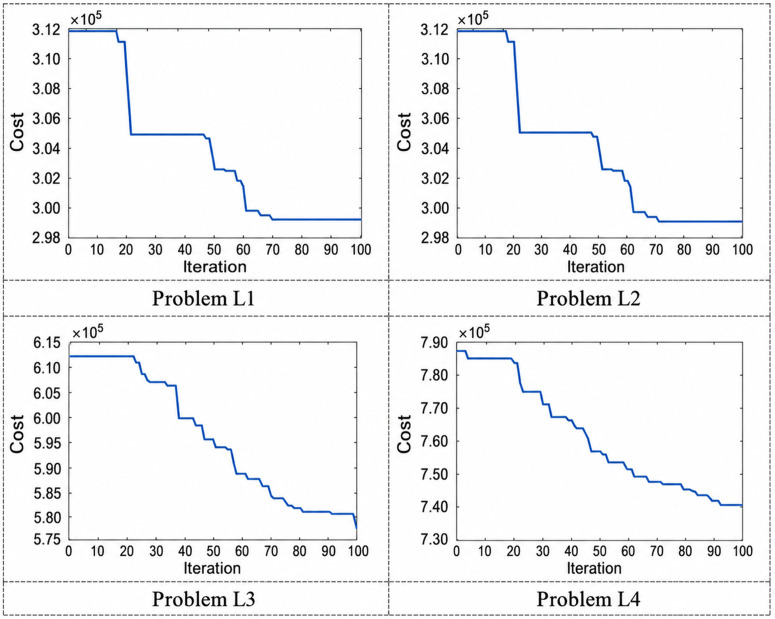
The convergence curve for large problems.

The solution of the model at this stage is carried out in four different dimensions. In the first three dimensions, as will be demonstrated later, the solution remains feasible in both software applications and with both precise and meta-heuristic approaches. The results also confirm the model’s performance, showing a very low gap. However, in the fourth dimension, the output from GAMS indicates that solving the model accurately using GAMS is not possible. Therefore, from this point onward, the model can only be solved using a genetic algorithm, which yields a reasonable result. Table 3 shows the results, and Fig 8 represents the convergence for large problems solved.

**Table 3:** The solution results for large problems.

Problem Dimension	Example Number	Number of								Solution Approach						
		Production Center	Vaccine	Distribution Center	Injection Center	Time Period	Binary Variable	Integer Variable	Constraint	GAMS		GA				Gap%
										Optimal Objective Function	CPU Time (s)	Population Size	Number of Generation	Best Objective Function	Standard Deviation	
**Larged-sized**	13	16	16	16	16	4	528	49152	4752	296529	613	1000	250	299268	965.1	0.0092
	14	20	20	20	18	4	820	92800	7220	408514	1020	1000	250	417307	1252.5	0.0215
	15	20	20	20	20	5	820	120000	9020	569153	1762	1000	250	578808	1365.54	0.017
	16	25	25	25	20	5	1275	218750	13400	–	–	1000	250	739539	1469.2	–

### Comparisons

**Fig 9:**
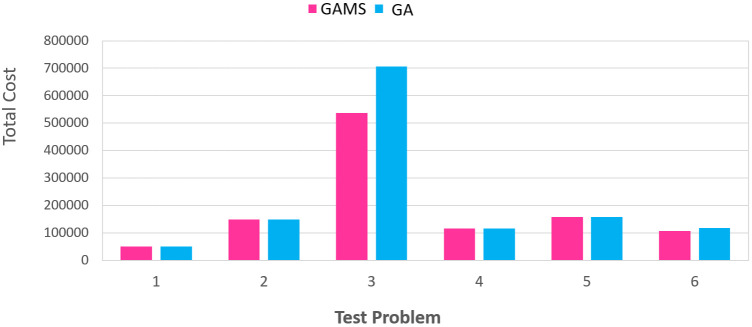
The comparisons for small problems.

**Fig 10:**
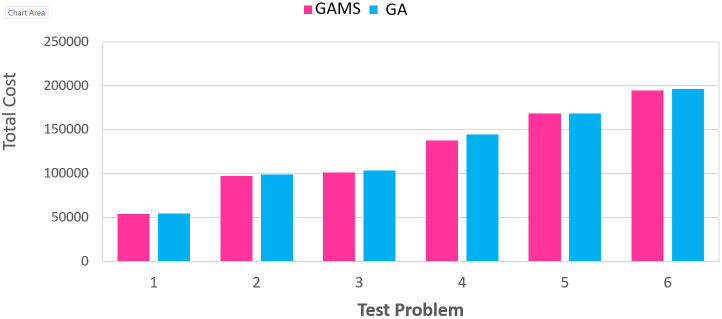
The comparisons for medium problems.

**Fig 11:**
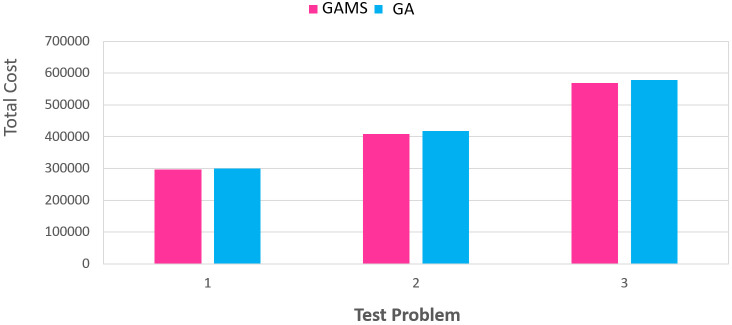
The comparisons for large problems.

**Fig 12:**
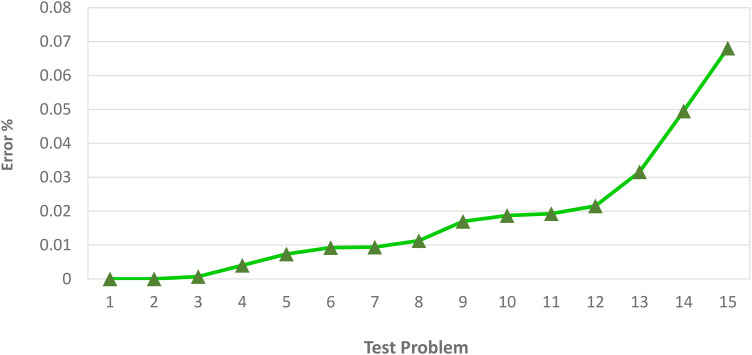
The error percentages for all problems.

As mentioned in the previous section, comparing the numerical results obtained from solving the model using two methods, GAMS (a precise method) and a genetic algorithm (an approximate heuristic method), and observing the gap between them indicates that the accuracy of the solution with the genetic algorithm is quite high. To facilitate a better understanding of this comparison, this section will utilize visual charts (Figs 9–12) to examine and compare the results from both approaches. In this fugures, the red points represent GAMS results, while the blue points denote GA outputs.

The performance of the proposed genetic algorithm was evaluated through multiple independent runs for each test problem in order to assess the consistency of the obtained solutions. For every problem instance, the algorithm was executed 10 times with different randomly generated initial populations, and the best objective function value together with the standard deviation was recorded. The relatively small standard deviation values reported in Tables 1–3 indicate that the proposed GA is capable of producing stable and high-quality solutions across different runs and problem scales.

The parameter settings of the genetic algorithm were selected based on preliminary computational experiments and convergence analyses. Different combinations of population size and number of generations were tested to achieve a balance between computational time and solution quality. According to the problem dimensions, the population size was varied between 50 and 1000, while the number of generations ranged from 20 to 250. The convergence behaviors illustrated in Figs 6–8 demonstrate that the algorithm reaches stable near-optimal solutions within an acceptable number of generations, confirming the effectiveness of the adopted parameter settings.

### Sensitivity analysis

In this section, a sensitivity analysis of the main parameters of the problem will be conducted. Accordingly, the changes in the objective function will be examined in relation to the modifications made to each parameter, ensuring the correct performance of the model.

**Fig 13:**
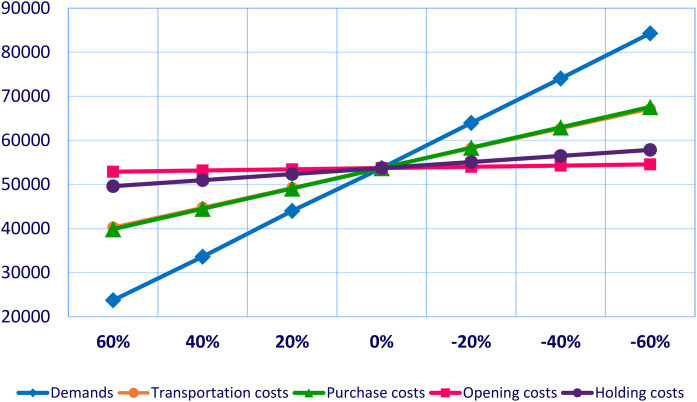
Sensitivity analysis trends.

Table 4 and Fig 13 present the results of the sensitivity analysis conducted to evaluate the robustness of the model with respect to variations in key parameters, including demand, transportation costs, purchase costs, opening costs, and holding costs. The percentage changes range from −60% to +60% relative to the baseline scenario.

**Table 4:** Sensitivity analysis results.

Parameter change (%)	Demands	Transportation costs	Purchase costs	Opening costs	Holding costs
−60%	84291.753	67156.050	67589.961	54569.434	57847.779
−40%	74059.736	62681.078	62970.353	54290.001	56475.565
−20%	63963.108	58206.107	58350.744	54010.569	55103.350
0%	53731.136	53731.136	53731.136	53731.136	53731.136
+20%	44004.294	49256.164	49111.527	53451.703	52358.921
+40%	33606.315	44781.193	44491.919	53172.270	50986.707
+60%	23735.786	40306.222	39872.310	52892.837	49614.492

As illustrated, the objective function value exhibits markedly different sensitivities across parameters. Demand emerges as the most influential factor: a + 60% increase in demand reduces the objective value significantly (to 23,735.786), whereas a −60% decrease leads to a substantial increase (up to 84,291.753). This inverse relationship indicates that the system benefits strongly from higher demand levels, likely due to improved utilization of network capacities and economies of scale.

Transportation and purchase costs show a moderate and nearly symmetric impact on the objective value. As these costs decrease, the total cost correspondingly declines, reflecting their direct contribution to operational expenses. However, their sensitivity is less pronounced compared to demand, suggesting that the model is relatively more resilient to fluctuations in these cost components.

In contrast, opening and holding costs demonstrate limited sensitivity. Variations in these parameters result in relatively small changes in the objective value, as also evident from the nearly flat trends in Fig 13. This indicates that fixed infrastructure decisions (opening costs) and inventory-related decisions (holding costs) are less critical drivers of overall system performance within the tested range.

The graphical representation in Fig 13 reinforces these observations by clearly showing the steep slope associated with demand changes, moderate slopes for transportation and purchase costs, and relatively flat lines for opening and holding costs. These findings highlight that demand uncertainty is the primary source of variability in the model, and thus, accurate demand estimation is crucial for achieving optimal system performance.

## Discussion

The results obtained from this study provide both quantitative and qualitative insights into the design and performance of the proposed vaccine supply chain network. From a quantitative perspective, the computational experiments clearly demonstrate the scalability challenges of the exact optimization approach. As shown in Tables 2–4, while GAMS is able to obtain optimal solutions for small- and medium-sized instances, the computational time increases significantly with problem size, reaching up to 1762 seconds for larger instances and eventually becoming infeasible for the largest case. In contrast, the proposed genetic algorithm (GA) consistently provides high-quality solutions with relatively small optimality gaps. Specifically, for small-sized problems, the gap is often zero or negligible, while for medium and large instances, it remains within an acceptable range (generally below 5%). This indicates that the GA is capable of maintaining solution quality while significantly reducing computational effort.

From a convergence standpoint, the results illustrated in Figs 6–8 show that the GA exhibits stable convergence behavior across different problem sizes. The objective function values decrease rapidly in early generations and gradually stabilize, indicating effective exploration and exploitation of the solution space. This performance confirms the suitability of the proposed chromosome structure and genetic operators in guiding the search process.

Qualitatively, the model offers several important managerial insights. First, integrating location, allocation, and storage decisions in a single framework leads to more efficient coordination across the supply chain. The results suggest that improper configuration of storage facilities (cold vs. ultra-cold) can significantly increase total costs, highlighting the importance of aligning infrastructure decisions with vaccine characteristics.

Second, the sensitivity analysis provides valuable insights into system behavior. As illustrated in Fig 13, the total cost increases in a consistent and nearly linear manner with respect to demand growth and cost coefficients. This confirms the logical consistency and robustness of the model. Moreover, the rate of cost increase suggests that demand surges during pandemics can lead to disproportionately higher logistics costs, emphasizing the need for proactive capacity planning.

Third, the results highlight the importance of distribution center selection and capacity allocation. Efficient placement and equipment of distribution centers reduce transportation and storage costs while ensuring demand satisfaction. The model demonstrates that allowing flexibility in equipping distribution centers with both cold and ultra-cold storage enhances adaptability and reduces system-wide costs.

From a practical perspective, the superiority of the proposed approach lies in its ability to balance realism and computational efficiency. Unlike many existing studies that simplify the problem by considering single-product or single-period settings, this model captures key real-world complexities, including multiple vaccine types, heterogeneous storage requirements, and time-dependent demand. At the same time, the GA-based solution approach ensures that the model remains applicable to large-scale, real-world problems where exact methods fail.

## Conclusions

This study developed a multi-product, multi-period vaccine supply chain network design model that simultaneously considers distribution center location decisions, vaccine allocation, and heterogeneous storage requirements, including cold and ultra-cold refrigeration systems. The proposed mixed-integer linear programming model was designed for a three-tier supply chain consisting of production centers, distribution centers, and injection centers, while ensuring that no shortage occurs at injection centers.

Because of the large-scale nature of the problem, a tailored genetic algorithm (GA) was developed to efficiently solve the model. The proposed GA incorporates a customized chromosome representation, feasibility-preserving initialization mechanism, crossover and mutation operators, and a reverse allocation procedure for determining vaccine flows throughout the network.

The computational experiments demonstrated the effectiveness of the proposed approach across small-, medium-, and large-sized problem instances. In small-sized problems, the GA achieved optimal or near-optimal solutions with very small gaps compared to GAMS. For example, in Problems 1 and 2, the GA reached the exact optimal solutions with 0% gap, while in Problem 4 the gap was only 0.08%. In medium-sized instances, the GA continued to provide high-quality solutions with acceptable deviations from optimality, where the gap remained below 5% in all tested cases. For instance, in Problem 10, the GA obtained a solution with a gap of 4.95% relative to the exact solution. Furthermore, in large-scale problems, the exact solution time increased significantly, reaching 1762 seconds in Problem 15, while GAMS was unable to solve the largest instance (Problem 16). In contrast, the proposed GA successfully generated a feasible high-quality solution with an objective value of 739,539 within reasonable computational effort. These results confirm the scalability and robustness of the proposed metaheuristic approach for solving complex vaccine supply chain planning problems.

The convergence analyses presented in Figs 6–8 also showed stable behavior of the GA across different problem dimensions. The algorithm rapidly improved solution quality during early generations and gradually converged toward stable near-optimal solutions, indicating an effective balance between exploration and exploitation mechanisms.

The sensitivity analysis further validated the consistency and practical applicability of the model. The results showed that demand is the most influential parameter affecting the objective function. Specifically, a 60% decrease in demand increased the total cost to 84,291.753, whereas a 60% increase reduced the objective value to 23,735.786. Transportation and purchasing costs exhibited moderate impacts on the objective function, while opening and holding costs showed comparatively lower sensitivity. These findings emphasize the importance of accurate demand forecasting and efficient infrastructure planning in vaccine distribution systems.

From a managerial perspective, the proposed framework can assist healthcare planners and policymakers in improving vaccine distribution efficiency under different storage technologies and operational conditions. The integration of cold and ultra-cold storage decisions within a unified optimization framework increases the flexibility of the supply chain and improves preparedness during large-scale vaccination campaigns and public health emergencies.

Future studies may extend the proposed model by incorporating advanced mathematical frameworks inspired by recent developments in optimal control, stochastic systems, dynamic optimization, and game theory. In particular, integrating stochastic demand evolution, delay-dependent vaccine deterioration, and dynamic feedback control policies could improve the responsiveness of vaccine supply chains during rapidly evolving pandemics.

## Supporting information

S1 File GAMS. (TXT)
